# A three-dimensional block structure consisting exclusively of carbon nanotubes serving as bone regeneration scaffold and as bone defect filler

**DOI:** 10.1371/journal.pone.0172601

**Published:** 2017-02-24

**Authors:** Manabu Tanaka, Yoshinori Sato, Hisao Haniu, Hiroki Nomura, Shinsuke Kobayashi, Seiji Takanashi, Masanori Okamoto, Takashi Takizawa, Kaoru Aoki, Yuki Usui, Ayumu Oishi, Hiroyuki Kato, Naoto Saito

**Affiliations:** 1 Department of Orthopaedic Surgery, Shinshu University School of Medicine, Asahi 3-1-1, Matsumoto, Japan; 2 Graduate School of Environmental Studies, Tohoku University, Aoba 6-6-20, Aramaki, Aoba-ku, Sendai, Japan; 3 Institute for Biomedical Sciences, Interdisciplinary Cluster for Cutting Edge Research, Shinshu University, Asahi 3-1-1, Matsumoto, Japan; 4 Department of Applied Physical Therapy, Shinshu University School of Health Sciences, Asahi 3-1-1, Matsumoto, Japan; 5 Aizawa Hospital Sports Medicine Center, Honjou 2-5-1, Matsumoto, Japan; University of Connecticut Health Center, UNITED STATES

## Abstract

Many recent studies have been conducted to assess the ability of composite materials containing carbon nanotubes (CNTs) with high bone affinity to serve as scaffolds in bone regenerative medicine. These studies have demonstrated that CNTs can effectively induce bone formation. However, no studies have investigated the usefulness of scaffolds consisting exclusively of CNTs in bone regenerative medicine. We built a three-dimensional block entity with maximized mechanical strength from multi-walled CNTs (MWCNT blocks) and evaluated their efficacy as scaffold material for bone repair. When MWCNT blocks containing recombinant human bone morphogenetic protein-2 (rhBMP-2) were implanted in mouse muscle, ectopic bone was formed in direct contact with the blocks. Their bone marrow densities were comparable to those of PET-reinforced collagen sheets with rhBMP-2. On day 1 and day 3, MC3T3-E1 preosteoblasts were attached to the scaffold surface of MWCNT blocks than that of PET-reinforced collagen sheets. They also showed a maximum compression strength comparable to that of cortical bone. Our MWCNT blocks are expected to serve as bone defect filler and scaffold material for bone regeneration.

## Introduction

In the event of a major bone defect due to bone trauma, bone tumor resection, osteolysis around joint prosthesis, and other factors, artificial bone repair is needed because bone tissue’s capacity to repair itself is limited. Traditionally, autologous or allogeneic bone transplantation has been performed to this end; however, both autologous and allogeneic transplantation pose some problems, including pain and nerve injury at the donor bone collection sites [1], limited amounts of bone collected for autologous bone transplantation, likely disease transfer [2], and high cost [3]of the procedure. For these reasons, there are expectations that bone regenerative medicine will eventually supplant bone transplantation methods.

Due to advances in regenerative medicine technology, tissue regeneration can now be induced using cells, growth factors, and scaffolds. Among these three factors of regenerative medicine [4], scaffolds has been attracting considerable attention and this attention has led to the development of a wide variety of scaffolds for bone and cartilage regeneration [5].

Many materials, including bioabsorbable polymers, ceramics, metals, and composite materials, have been investigated as scaffold materials for bone regenerative medicine [6], and the number of studies investigating the scaffold use of carbon nanotubes (CNTs) has been increasing [7]. In bone regenerative medicine, in particular, a CNT/polylactic acid complex was shown, in 2002, to promote osteoblast proliferation *in vitro* [8]. Subsequently, reports revealed strong adhesion of osteoblasts to CNT/polycarbonate urethane complex and CNT/poly-lactic-co-glycolic acid complex [9][10]. In 2006, CNTs (when used alone) were shown to promote the proliferation of osteocytes and osteoblasts [11]. A number of *in vitro* studies were then published pointing out the special effects of CNTs on bone-related cells [12][13]. In 2008, we showed that CNTs also promoted bone tissue formation *in vivo* for the first time [14][15][16]. Specifically, bone tissue was formed in the mouse back muscle earlier when using a composite of a collagen sheet compounded with multi-walled CNTs (MWCNTs) as a carrier for rhBMP-2, than when using a collagen sheet scaffold alone. Thereafter, many investigators reported that CNTs promoted bone regeneration *in vivo* or *in vitro*, raising expectations for their application as scaffold material in bone regenerative medicine[11][16].

To date, however, no investigators have built a three-dimensional structure of CNTs alone for use as a scaffold in bone regenerative medicine. We prepared a block entity with maximized mechanical strength by three-dimensionally building up MWCNT blocks [17][18]. In this study, the osteoinductive performance of our scaffold (consisting of three-dimensional MWCNT blocks and rhBMP-2) was assessed using an *in vivo* ectopic bone formation test and an *in vitro* test of the proliferative activity of three types of cells. RhBMP-2 is used not only in basic research but also in clinical treatment, and collagen sheets are the gold standard vehicle for delivering drugs. We used PET-reinforced collagen scaffolds as non-shrinking controlled-release drug delivery systems [19][20]. As far as we know, this study is the first to describe the application of CNTs alone as a scaffold in regenerative medicine and to show the clinical potential of CNTs in regenerative medicine not only for bone tissue but also many other tissues.

## Materials and methods

### MWCNT block

MWCNTs were obtained from NanoLab Inc. (Waltham, MA, USA) and synthesized by the chemical vapor deposition method. The purity of the MWCNTs was about 80 wt%, and the rest of the material consisted of amorphous carbon, Al, Fe, Mo, and Cr. The nanotubes exhibited a bamboo-like morphology, with average diameter 20–40 nm and length 500 nm–5.0 μm. MWCNT powder was burned in air at 500°C for 90 min in order to remove amorphous carbons. At this point, 1.0 g of the burned sample was then introduced into a flask containing 6 mol/L HCl to dissolve the Fe, Mo, and Cr. Following this, the acid solution was filtered, and 0.5 mg of the washed soot was transferred to a flask with 0.5 L of 6.8 mol/L HNO_3_ and refluxed at 100°C for 16 h. The resulting suspension was filtered and washed with distilled water. Finally, the samples were dried *in vacuo* at 100°C for 24 h. The carboxylated MWCNTs were thereafter referred to as “COOH-MWCNTs”. After this treatment, impurities in this sample were determined to be Al (0.16 wt%), Fe (0.32 wt%), and Mo (0.02 wt%) by inductively coupled plasma-optical emission spectrometry. MWCNT blocks were prepared using a spark plasma sintering system (SPS-1050, Sumiseki Materials, Tokyo, Japan). Typically, 0.3 mg of the COOH-MWCNT powder was used and hardened in a graphite covered die with a diameter of 20 mm at 1000°C (heating rate, 25°C/min) under a pressure of 80 MPa *in vacuo* (1 x 10^2^ Torr) for 10 min. The resulting blocks were then processed further (i.e., cut and polished). These materials and methods were already published [17][18].

### Characteristics of MWCNT blocks

**Fig 1:**
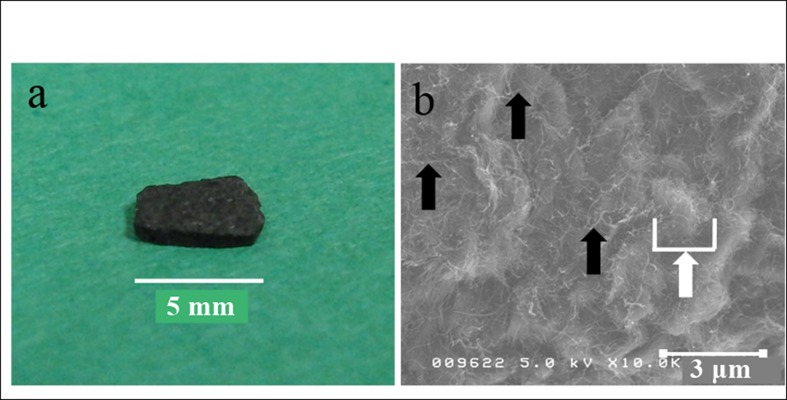
MWCNT block. (a) A macroscopic image of an MWCNT block. (b) Scanning electron photomicrograph of an MWCNT block. Black arrow: nanosize irregularity formed by MWCNT fibers. White arrow: microsize irregularity formed by an aggregate of MWCNT fibers.

A macroscopic image and scanning electron photomicrograph of an MWCNT block used in this study are presented in Fig 1. The sample morphologies were determined by SEM (S-4100, Hitachi, Japan) operated at 5kV. The blocks were found to have nanosize irregularities due to CNT fibers on their surfaces, which (when combined) formed microsize irregularities. MWCNT blocks possessed a microsize concave-convex surface with nanotubes. The contact angle of the MWCNT blocks was 27.8° SD (S.D. ± 7.7), which reflecting the high hydrophilicity of their surfaces. Table 1 shows the morphological and mechanical properties of MWCNT blocks and cortical bone. The density and Vickers yield strength of MWCNT blocks were comparable to those of cortical bone [21][22][23]. On the other hand, Young’s modulus of MWCNT blocks was about 1/2 to 2/3 that of cortical bone, and their bending strength (29.0–47.4 σb) was about 1/4 to 1/3 that of cortical bone.

**Table 1:** A comparison of the mechanical characteristics of MWCNT blocks and cortical bone.

Characteristic	MWCNT blocks*	Cortical bone	Ref.
Bulk density, ρ (g/cm^3^)	1.34	1.304–1.882	[23]
Young's modulus, E_b_ (Gpa)	6.4–7.8	9.82–15.7	[22]
Fracture bending strength, (σ_b_)	29.0–47.6	103.5–238.2	[22]
Vickers hardness, H_v_ (Mpa)	33.2	31.6–34.2	[21]

* Adapted with permission from Sato Y, Ootsubo M, Yamamoto G, Van Lier G, Terrones M, Hashiguchi S, et al. Super-robust, lightweight, conducting carbon nanotube blocks cross-linked by de-fluorination. ACS Nano. 2008;2: 348–356. Copyright 2008 American Chemical Society.

### Mechanical testing

Uniaxial compression tests were carried out on both of the MWCNT blocks (cut into round pieces of 3 mm in diameter and 1 mm in height), poly(ethylene terephthalate) (PET)-fiber-reinforced collagen sheets (MedGEL, MedGEL, Kyoto, Japan), and rat femoral bones(n = 5). Bones dissected from femoral shafts of 10-week-old male Wistar rats (SLC, Shizuoka, Japan) were cut into cylinders of 3 mm in diameter and 1 mm in height. Compressive strength was measured in a direction perpendicular to the bottom surface of each cylinder using an Autograph AGS-H (Shimadzu Co, Ltd, Kyoto, Japan) at compression speed of 1 mm/min.

### Qualitative determination of protein adsorption profiles

MWCNT blocks, PET-fiber-reinforced collagen sheets, and coverslips (5 mm in diameter; Matsunami Glass, Osaka, Japan) (n = 5) were immersed in 500 μL of Bovine Serum Albumin (BSA) solution (500 μg/mL, Wako Pure Chemical Industries, Osaka, Japan) for four days. Subsequently, protein concentrations of these solutions were analyzed using a protein assay BCA kit (Nacalai Tesque, Kyoto, Japan).

### Protein loading efficiency

Ten μL of 40 mg/mL BSA solution was dropped (400 μg/sample) onto MWCNT blocks and PET-fiber-reinforced collagen sheets (n = 5) in wells of a 48-well plate. The scaffolds were dried for 24 hours and removed from the wells. Then the protein concentration in a supernatant consisting of the remaining BSA dissolved in 1 mL of sterilized water was determined using a protein assay BCA kit (Nacalai Tesque). The protein loading efficiency was calculated by the following equation:

$$Loading\;efficiency\;(\%)=\frac{M_{actual}}{M_{theoretical}}=\frac{400-M_{remaining}}{400}$$

where *M*_actual_ is the actual amount of BSA binding to the MWCNT blocks as determined by the above experiment, *M*_theoretical_ is the theoretical amount of BSA bound (400 μg), and *M*_remaining_ is the amount of BSA remaining in the wells after block adsorption.

### *In vitro* protein releasing assay

Twenty μL of 50 mg/mL BSA solution was dropped (1000 μg/sample) onto MWCNT blocks and PET-fiber-reinforced collagen sheets (n = 5). The scaffolds were dried and immersed in 500 μL of sterilized water. Supernatants were retrieved on day 0 (1 hour), 1, 3, 5, 7, 10, 14, 17, and 21, and then new sterilized water was added. The protein concentration of each supernatant was analyzed using a protein assay BCA kit (Nacalai Tesque).

### Ectopic bone formation stimulated by BMP on MWCNT blocks serving as a scaffold

MWCNT blocks, cut into round pieces (5 mm in diameter, 1 mm in height), were sterilized by immersion in 99.5% ethanol (a widely used surface-sterilizing agent) [24][25] for 1 h and dried in an incubator for 24 h. To prepare the scaffolds containing BMP-2, a solution of rhBMP-2 (5 μL of 1 mg/mL; Peprotech EC, London, UK) was dropped onto each scaffold, which was then dried at 37°C for 24 h. Separately, a PET-fiber-reinforced collagen sheet (MedGEL) of the same size, after sterilization in the same manner, and treatment with the same amount of rhBMP-2 was used as a positive control [26][22]. The back skin and fascia of each 6-week-old male ddY mouse (SLC, Shizuoka, Japan) were incised, and MWCNT blocks (for 7 animals) or BMP-2-containing PET-fiber-reinforced collagen sheets (for another 7 animals) were aseptically inserted into the back muscle, and the skin was sutured. At 3 weeks postoperatively, mice were euthanized by isoflurane anesthesia and subjected to micro-computed tomography (μCT) (R mCT, Rigaku, Tokyo, Japan) (conditions: tube voltage 90.0 kV, tube current 40.0 μA). The resulting images were reconstituted using the i-View software (J. Morita MFG, Kyoto, Japan) and analyzed using ImageJ software (http://imagej.nih.gov/ij/) [27]. The bone mineral density (BMD) of the ectopic bone formed in each mouse was measured using the quantitative computed tomography (qCT) technique. Slices containing ectopic ossification were selected using μCT, and a region of interest (ROI) was defined in the ectopic ossification area on each slice. CT values for all ROIs were measured and averaged to obtain a CT value for each mouse. Thereafter, the CT values were corrected using the model UHA phantom for bone mineral quantification (hydroxyapatite concentrations: 0, 100, 200, 300, and 400 mg/cm^3^) (Kyoto Kagaku, Kyoto, Japan), and they were converted to g/cm^3^ values (n = 7). These images were rendered to 3D by VG Studio MAX software (Volume Graphics GmbH, Heidelberg, Germany), and total volume, bone volume, BV/TV (bone volume to total volume), BS/BV (bone surface to bone volume), TbTh (mean trabecular thickness), TbN (mean trabecular number), TbSp (mean trabecular spacing) were subsequently evaluated from these 3D images. After that, the compression strength and energy to failure of the scaffold were measured by Autograph AGS-H (SHIMADZU Co., Kyoto, Japan).

The MWCNT block and PET-fiber-reinforced collagen sheet were each taken out en bloc with surrounding soft tissue. After fixation with a 20% neutrally buffered formalin solution (Wako Pure Chemical Industries, Osaka, Japan), each tissue specimen was embedded using Osteoresin (an embedding kit; Wako Pure Chemical Industries, Osaka, Japan), sectioned using a saw microtome (SP1600, Leica AG, Glattbrugg, Switzerland), stained with hematoxylin-eosin, and then examined under a light microscope. Trabeculae containing osteocytes (intensely stained by eosin, with nuclei intensely stained by hematoxylin) and hematopoietic marrow within trabecullar bone (nucleus stained purple by hematoxylin, cytoplasm stained pink by eosin) [28][29] were examined. All experimental protocols were approved by the institutional animal care committee of Shinshu University. The methods were carried out in accordance with the approved guidelines.

### Cell culture and seeding onto the scaffold

MC3T3-E1 preosteoblasts[30] (RIKEN Cell Bank, Tsukuba, Japan) were cultured in regular culture media consisting of alpha-modified minimum essential medium (alpha-MEM; Nacalai Tesque) supplemented with 10% heat-inactivated fetal bovine serum (GIBCO, Grand Island, NY, USA) and 1% Antibiotic‐Antimycotic Mixed Stock Solution (Nacalai Tesque) in a humidified atmosphere of 5% CO_2_ at 37°C. The cells were trypsinized and seeded onto sample scaffolds at 4 x 10^4^cell /scaffold before each experiment. In all experiments, cells (20 μL of each cell suspension at a suitable cell concentration) were added to each scaffold using a micropipette and incubated in a 5% CO_2_ saturated humidity incubator for an hour at 37°C until the cells adhered to the scaffold. Thereafter, each scaffold was transferred to a plate (size suitable for each experiment) and cultured in an appropriate amount of alpha-MEM with medium replacement twice a week.

### Test for cell proliferation on MWCNT blocks

MWCNT blocks, cut into 2-mm cubes, were sterilized by immersion in 99.5% ethanol for 1 h and dried in an incubator for 24 h. PET-fiber-reinforced collagen sheets (MedGEL, Kyoto, Japan) of the same size were used as a positive control. MC3T3-E1 cells (mouse preosteoblasts; RIKEN Cell Bank) were maintained at 37°C in a 5% CO_2_ incubator, cultured in α-MEM medium supplemented with 10% fetal bovine serum (FBS), and passaged twice a week. MWCNT blocks, collagen sheets, and hydroxyapatite discs (HA disc, 3D Biotek, NJ, USA) (n = 5) were placed in 48-well plates and seeded with cells of each type at 4 x 10^4^ cells/scaffold according to the above-described method, and cultured in the presence or absence of 100 ng/ml rhBMP-2 for 21 days. The media were replaced twice a week. On day 1, 7, and 21, the cells were stained with bisbenzimide H33342 fluorochrometrihydrochloride (Hoechst 33342, Nacalai Tesque, Kyoto, Japan) and counted. For each sample, one randomly selected portion was photographed under a fluoroscence microscope (200-fold magnification). Using an image analyzer (DP2-BSW software, Olympus, Tokyo, Japan), cells in one visual field were counted, and the mean counts for seven scaffolds were compared. To compensate for the limited field depth of the 3D cell culture images, the images at multiple Z-levels were stacked using Zerene Stacker software (Zerene Systems, Richland, WA, US).

### Observation of cell adhesion by fluorescence microscopy

MC3T3-E1 cells were cultured for three days, fixed for an hour in 4% paraformaldehyde, treated an hour with 0.1% Triton-X, stained an hour with FITC-phalloidin (Sigma Aldrich, St. Louis, MO) to show F-actin (red) and bisbenzimide H33342 fluorochrome trihydrochloride (NacalaiTesque) to show nuclei (blue), washed in PBS (NacalaiTesque), and observed under a confocal laser scanning microscope (LSM 5 Exciter, Zeiss, Germany). Cell adhesion on the scaffold surface was evaluated.

### Alkaline phosphatase activity assay

MC3T3-E1 cells (1.0 × 10^4^ cells/scaffold) were seeded on MWCNT blocks and PET-reinforced collagen sheets (n = 3) in a 48-well plate and cultured in alpha-MEM in the presence or absence of 100 ng/ml of rhBMP-2 with a change of medium every 3 days. On day 21 of culture, the cells were washed with PBS twice and sonicated in lysis buffer consisting of 0.1% Triton X-100. After centrifugation at 10 000 × g for 5 min at 4°C, a sample of each supernatant was transferred to tubes for measurement of alkaline phosphatase (ALP) activity using a LabAssay ALP kit (Wako Pure Chemicals Industries, Osaka, Japan) and protein concentration using a BCA Protein Assay kit (Nakalai Tesque). The relative activity of the sample is reported as the ratio of activity to the corresponding protein concentration (μmol/g). Significant differences were identified by the Student *t* test.

### Statistical analysis

Data are tabulated as mean ± standard deviation values and were statistically analyzed using the Student *t* test and one-way ANOVA, followed by Tukey's post hoc test, with *P<0.05 considered to indicate a significant difference.

## Results

### Mechanical testing

**Fig 2:**
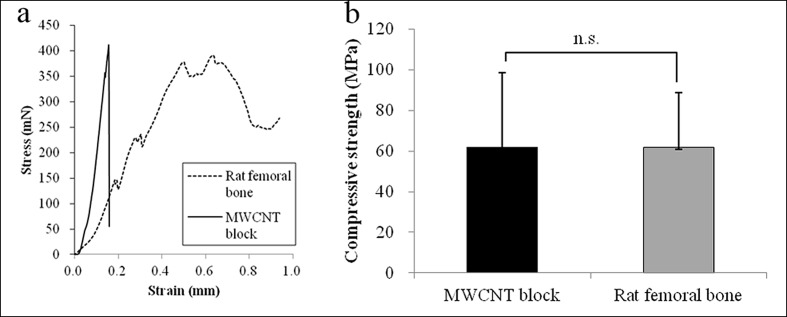
Compression test. (a) Compressive strength of both samples. (b) Representative load-displacement curves of MWCNT blocks and rat femoral bone specimens. Values are mean ± S.D.; n = 5.

Fig 2A shows representative load-displacement curves of MWCNT blocks and femoral bone specimens, respectively. It can be seen that the load at the yield point was higher for the MWCNT blocks than femoral bone cylinders, which fractured catastrophically without showing a no clear yield point. This finding shows that the fracture behavior of the MWCNT block differes from that of the femoral bone.

The maximum compressive strengths (in MPa) of the MWCNT blocks and the femoral bone specimens (62.06 ± 36.40 and 61.86 ± 26.88 respectively) were not significantly different (P = 0.9924, Fig 2B).

### Qualitative protein adsorption profiles

**Fig 3:**
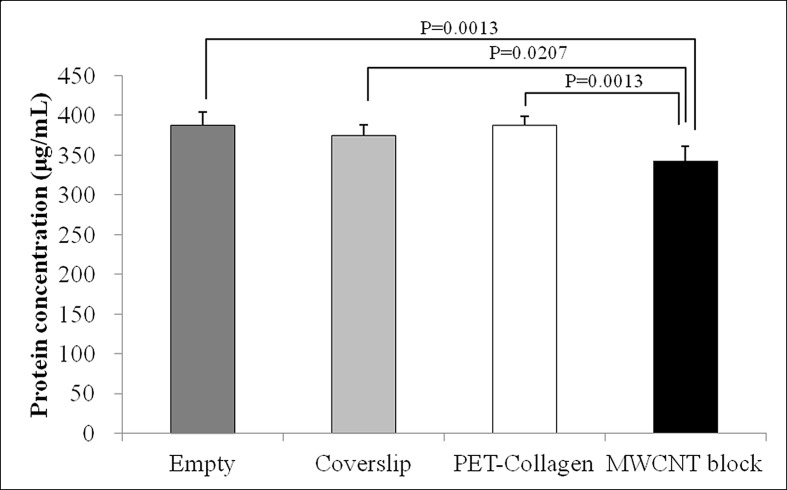
Protein adsorption. Qualitative Protein Adsorption Profiles of MWCNT blocks and PET-reinforced collagen sheets. Values are mean ± S.D.; n = 5.

On day 4, significantly more protein was adsorbed by MWCNT blocks than by PET-fiber-reinforced collagen sheets and Coverslips (*P* = 0.0207 and 0.0013, respectively). The total amounts of protein that adsorbed to MWCNT blocks, PET-fiber-reinforced collagen sheets, and Coverslips during 4 days were 57.0, 12.6, and 25.7 μg, respectively (Fig 3).

### Protein loading efficiency

**Fig 4:**
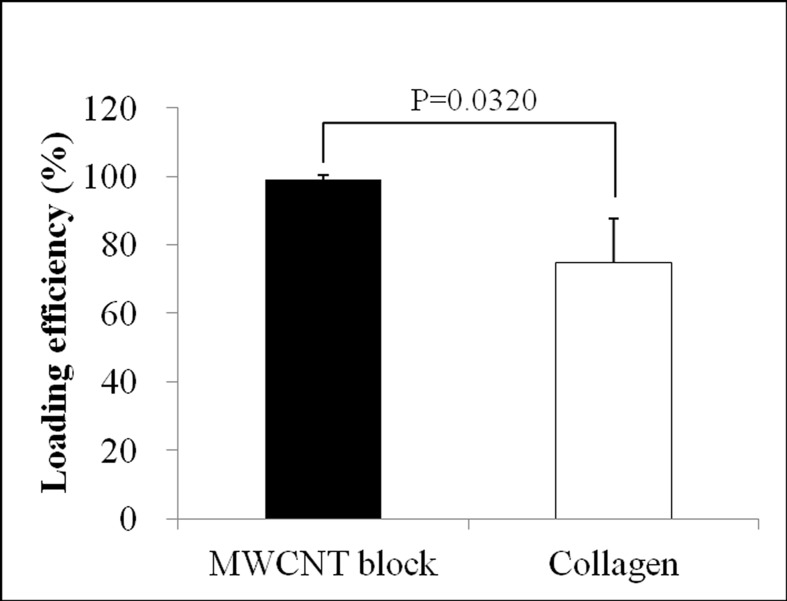
Protein loading efficiency. Graph shows the protein loading efficiency of MWCNT blocks and PET-reinforced collagen sheets. Values are mean ± S.D.; n = 5.

The protein loading efficiency of MWCNT blocks was high (93.98 ± 1.41%) and significantly higher than that of PET-reinforced collagen sheets (74.69 ± 12.99%) (Fig 4).

### *In vitro* protein releasing assay

**Fig 5:**
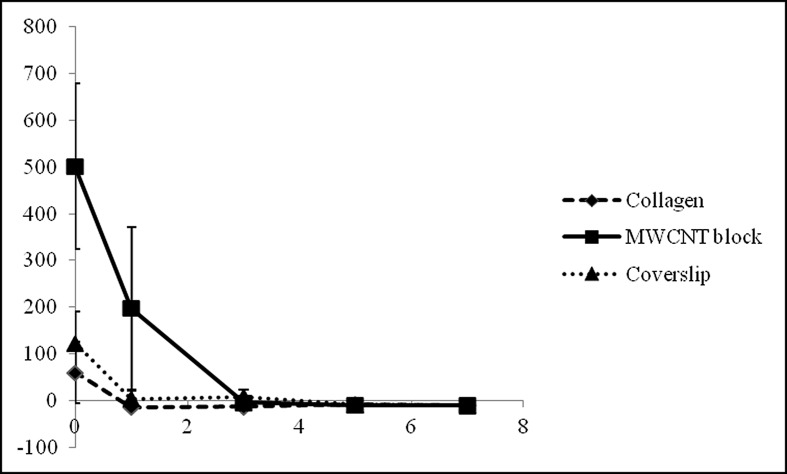
*In vitro* protein releasing assay. Graph shows the protein concentration of the supernatants in which MWCNT blocks, PET-reinforced collagen sheets and coverslips were immersed. Values are mean ± S.D.; n = 5.

The protein was released from both MWCNT blocks and PET-reinforced collagen sheets. On day 0, MWCNT blocks released more protein (501.36 ± 177.20 μg) than PET-reinforced collagen sheets (60.75 ± 65.21 μg). After day 1, MWCNT blocks kept releasing protein (198.38 ± 173.84 μg), whereas PET-reinforced collagen sheets released almost no protein (Fig 5).

### Ectopic bone formation stimulated by BMP on MWCNT block scaffolds

**Fig 6:**
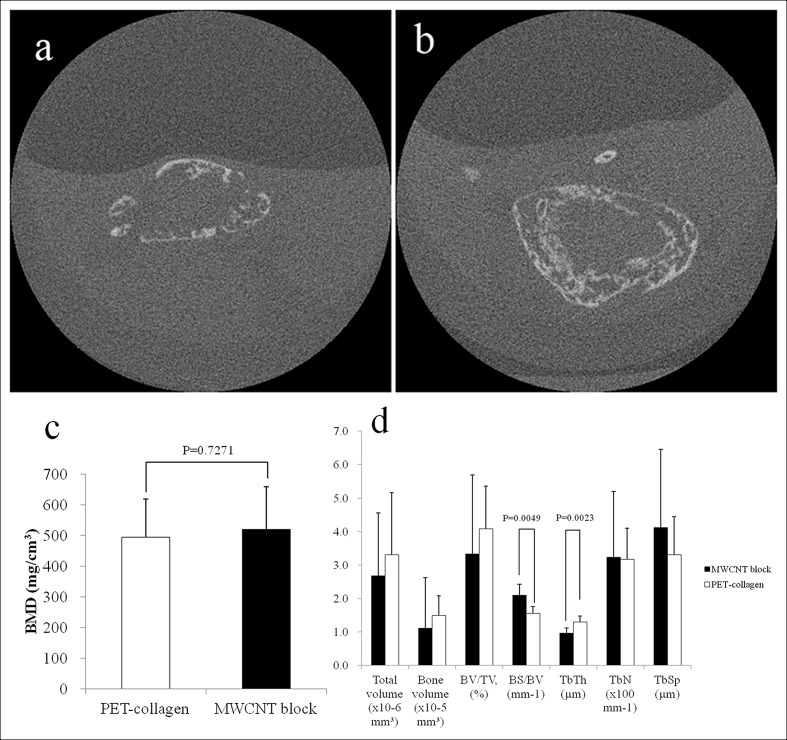
The μCT evaluation at 3 weeks after implanting rhBMP-2-containing scaffold in the mouse back muscle. (a) CT image of ectopic bone formed using a PET-fiber-reinforced collagen sheet (positive control) as a scaffold (arrow). (b) CT image of ectopic bone formed using MWCNT blocks. Distinct bone formation was observed in both cases. (c) BMD values of ectopic bone formed around an rhBMP-2-containing PET-fiber-reinforced collagen sheet and rhBMP-2-containing MWCNT block. (d) Three-dimensional μCT analysis. BV/TV: bone volume / total volume; BS/BV: bone surface / bone volume; TbTh: trabecular thickness; TbN: trabecular number; TbSp: trabecular spacing.

**Fig 7:**
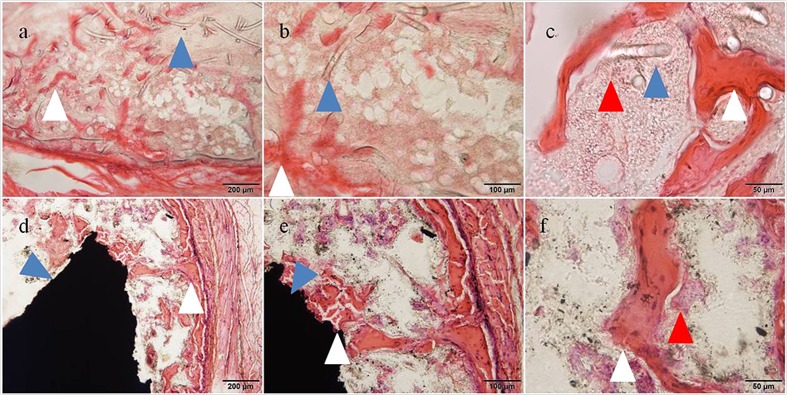
Histological images taken 3 weeks after implantation in mouse back muscle (Hematoxylin-eosin staining). (a, b, c) Ectopic bone formed by an rhBMP-2-containing PET-fiber-reinforced collagen sheet. Blue arrow: remnant of PET-fiber-reinforced collagen sheet. White arrow: Trabecular structure of newly formed bone. Red arrowhead: Bone marrow cells. (d, e, f) Ectopic bone formed by an rhBMP-2-containing MWCNT block. The newly formed bone was found to contain a small number of MWCNTs. Blue arrowhead: MWCNT block. White arrowhead: trabecular structure of newly formed bone. Red arrowhead: Bone marrow cells.

After cutting each MWCNT block into round pieces of 5 mm in diameter, 5 μg of rhBMP-2 was added. Separately, a PET-fiber-reinforced collagen sheet of the same size with the same amount of rhBMP-2 was used as a positive control. To evaluate ectopic ossification, an incision was made on the back of mouse to prepare a subfascial pocket in the back muscle, where an rhBMP-2-containing MWCNT block or an rhBMP-2-containing PET-fiber-reinforced collagen sheet was implanted. At 3 weeks postoperatively, mice were subjected to μCT. The resulting images were reconstituted using the i-View software and analyzed using ImageJ software [23]. The μCT evaluation revealed distinct ectopic bone formation. The ectopic bone that formed on MWCNT blocks containing 5 μg of rhBMP-2 was similar to that on the positive control PET-fiber-reinforced collagen sheet containing 5 μg of rhBMP-2 (Fig 6A and 6B). No bone formation was observed on those scaffolds without rhBMP-2. Fig 6C shows the bone mineral densities (BMDs) of ectopic bone formed using a PET-fiber-reinforced collagen sheet and MWCNT blocks as scaffolds (i.e., 495.3 mg/cm^3^ vs 520.6 mg/cm^3^ on average, respectively). Although the value was higher for the MWCNT block group, the difference was statistically insignificant. Total volume, bone volume, BV/TV, TbN, and TbSp were not significantly different between the two scaffolds. BS/BV was higher (2.1054 ± 0.3268 vs 1.5603 ± 0.2009, P = 0.0049) and TbTh was lower (0.9698 ± 0.1499 vs 1.3006 ± 0.1715, P = 0.0023) for the MWCNT blocks than the PET-fiber-reinforced collagen sheets (Fig 6D). Fig 7A shows representative stress-strain curves for the ectopic bones formed around MWNCT blocks and collagen sheets, in the presence and absence of rhBMP-2, respectively. MWCNT blocks with rhBMP-2 were more stress resistant and had a higher yield point than MWCNT blocks without rhBMP-2, while the PET-reinforced collagen sheets showed no clear yield point and exhibited non-brittle failure at a lower stress level than the MWCNT blocks, even if bone formed around it. This shows that the fracture behavior of bone around MWCNT blocks differs from that of the bone around PET-reinforced collagen sheets. The compressive yield strength after the initial breakdown was higher in the MWCNT block, and breaking energy showed a significant difference between the two scaffolds (3.04 ± 0.24 vs 2.51 ± 0.19 J, p = 0.0081 in the absence of rhBMP-2, 3.12 ± 0.30 vs 2.63 ± 0.08 J, p = 0.0158 in the presence of rhBMP-2, respectively) (Fig 7B).

**Fig 8:**
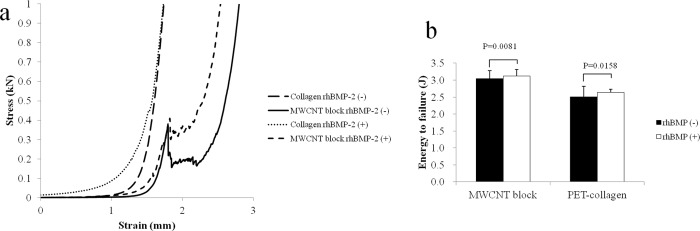
Compression test of ectopic bones formed around MWCNT blocks. Graph shows (a) the energy to failure and (b) the Stress-Strain curves of ectopic bones formed around the MWCNT blocks and PET-reinforced collagen sheets in the presense and absence of rhBMP-2.

Histological specimens were prepared from tissue samples extirpated three weeks after implantation from animals in the PET-fiber-reinforced collagen sheet group and those in the MWCNT block group. An evaluation using a light microscope revealed the degradation of the PET-fiber-reinforced collagen sheet and the formation of ectopic bone around its remnant. A trabecular structure with bone marrow tissue (cell nuclei stained purple by hematoxylin and cytoplasm stained pink by eosin) was observed (Fig 8A–8C). A trabecular structure with bone marrow tissue was also found around the MWCNT blocks, confirming the formation of ectopic bone. Newly formed bone was seen as bulging within the surrounding connective tissue. A trabecular structure was also found in areas in direct contact with MWCNT blocks, demonstrating a strong bond had developed between MWCNT blocks and newly formed bone. A small number of MWCNTs were observed in newly formed bone (Fig 8D–8F).

### Test for cell proliferation on MWCNT blocks

**Fig 9:**
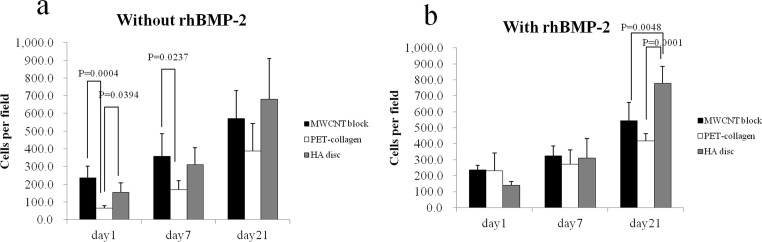
Proliferation of MC3T3-E1 cells on MWCNT blocks and PET-reinforced collagen sheets. Cell counts (mean ±S.D. *P<0.05) on PET-reinforced collagen sheets and MWCNT blocks in the (a) presence and (b) absence of rhBMP-2. Values are mean ± S.D.; n = 5.

On day 21 with the absence of rhBMP-2, no significant difference in cell proliferation between the MWCNT blocks, PET-reinforced collagens, and HA discs was observed (571 ± 157 vs 388 ± 154 cells per scaffold, P = 0.2934 and vs 680 ± 230 cells per scaffold, P = 0.6315, respectively). On day 1 and 7, a significant increase in cell number on the MWCNT blocks was observed compared to the PET-reinforced collagens (235 ± 65 vs 65 ± 13 cells per scaffold, p = 0.0004 and 358 ± 127 vs 170 ± 49 cells per scaffold, p = 0.0237) (Fig 9A).

On day 21 with the presence of rhBMP-2, a significant increase in cell number on the HA discs was observed compared to the MWCNT blocks and PET-reinforced collagens (777 ± 105 vs 417 ± 44 cells per scaffold, p = 0.0001 and vs 543 ± 113 cells per scaffold, p = 0.0048) (Fig 9B).

### Assessment of cell adhesion by fluorescence microscopy

**Fig 10:**
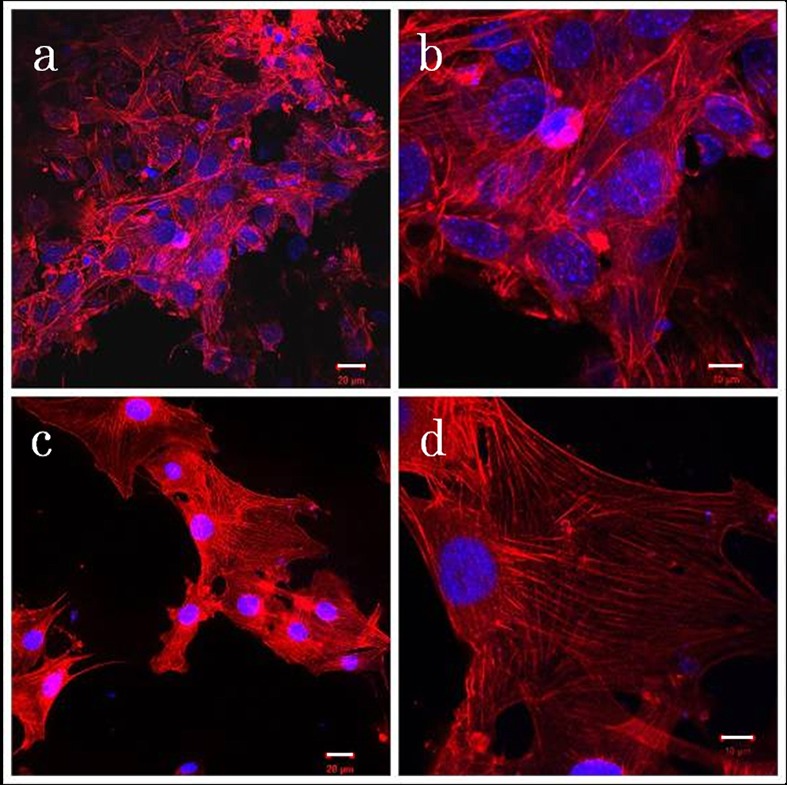
MC3T3-E1 cell morphology on MWCNT blocks. Fluorescence photomicrographs of cell cultures on PET-reinforced collagen sheets (a,b) and MWCNT blocks (c,d). White bars: 20μm for (a,c) and 10μm for (b,d).

Panels a to d in Fig 10 show fluorescence photomicrographs of cell cultures, demonstrating cell proliferation on PET-reinforced collagen sheets (a, b) and MWCNT blocks (c, d) on Day 3 of culture. Cell coverage was nearly complete on the MWCNT block surface but small on the PET-reinforced collagen, with some cells adhering to PET fibers. Morphological examination revealed, in both cases, polygonal cells having a large number of filopodia that connected the cell membrane to the scaffold surface. Stress fibers were observed in the cells.

### Alkaline phosphatase activity assay

**Fig 11:**
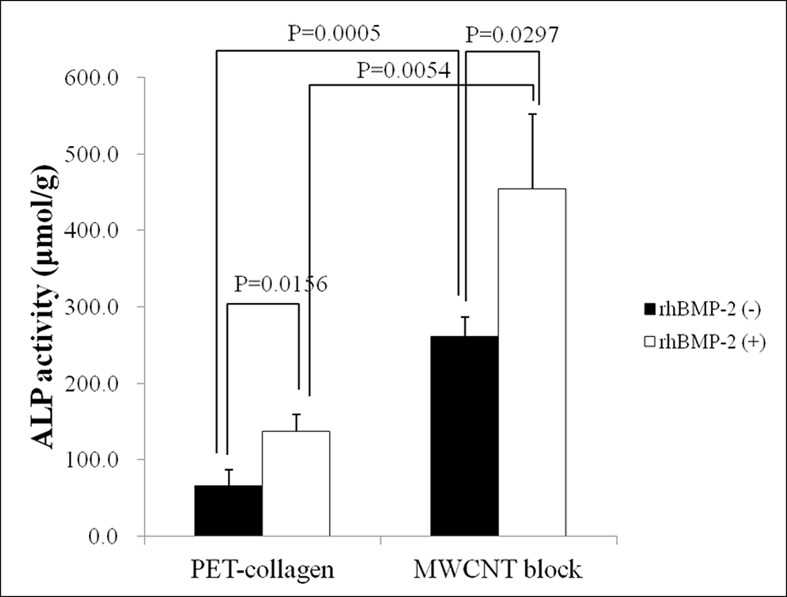
ALP activity. Graph shows the ALP activity of MC3T3-E1 cultivated on MWCNT blocks and PET-reinforced collagen sheets in the presence and absence of rhBMP-2. Values are mean ± S.D.; n = 3.

After 21 days, ALP activity was significantly higher in cells on MWCNT blocks than in cells on PET-reinforced collagen sheets in both the presence and absence of rhBMP-2 (P = 0.0005 and 0.0054, respectively) (Fig 11).

## Discussion

**Fig 12:**
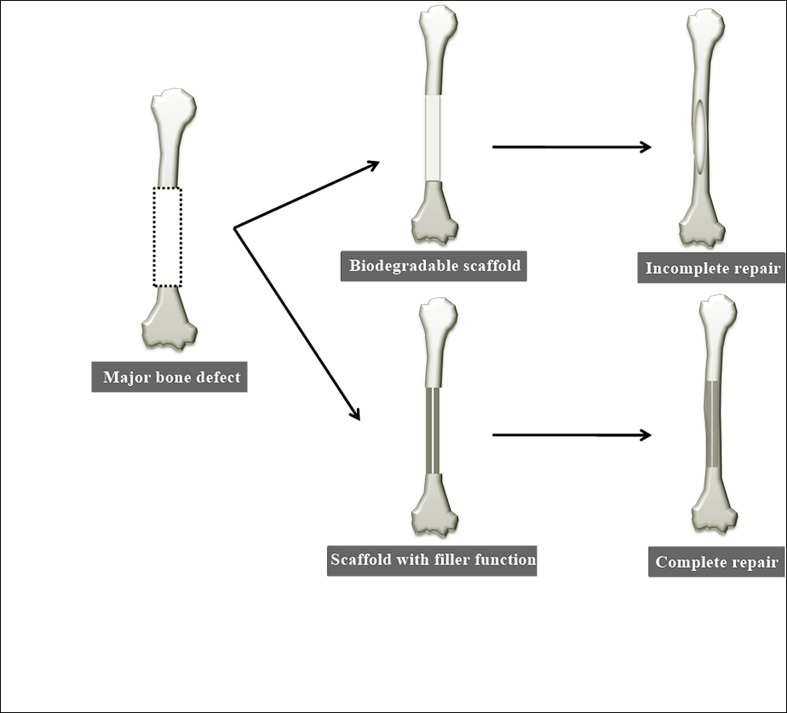
Scheme for bone regeneration treatment using a biodegradable scaffold and a scaffold that also serves as a filling material. There is a need for a non-biodegradable scaffold with the high osteoinductive potential of BMP and capacity to fill the gaps left by bone defects.

Scaffold characteristics reported to be essential for bone regenerative medicine include bioabsorbability, mechanical strength, growth factor retention and release capacity, and cellular bioavailability [6]. BMP as a growth factor and collagen sheets as bone-growth scaffolds are already in clinical application [31]. However, this therapeutic modality does not always yield favorable results [32], limiting its clinical application, with conventional bone transplantation often performed instead. One reason for this may be the necessity for large quantities of BMP to induce bone formation in clinical settings. In addition, the entire bone defect must be filled with newly formed bone and collagen sheets (though excellent scaffolds for BMP delivery) are biodegradable and no substitute for bone. In some cases, complete osteogenesis does not occur even after absorption of the bone graft and scaffold [33]. The same applies to other scaffolds that are biodegradable polymers [34]. For this reason, there is a need for a non-biodegradable scaffold with the high osteoinductive potential of BMP and capacity to fill the gaps left by bone defects [35]. Such a scaffold would reduce the amount of regenerated bone needed, thus reducing the requirement for BMP (Fig 12).

Our results demonstrate that MWCNT blocks serve as filler materials that carry rhBMP-2. MWCNT blocks are solid, having nanosized surface irregularities and substantially non-porous interiors. Therefore, cells cannot enter the scaffold. MWCNT is expected to function as an osteoconductive scaffold (by allowing osteoblasts to proliferate on the MWCNT block surface) and as a filler. Currently, other attempts to this end are being made by using hydroxyapatite (HA) and other materials as scaffolds [36,37]. However, ectopic bone formation cannot be induced merely by adding BMP to calcium phosphate; collagen must be available as a carrier for BMP [38]. We reported in 2008 that MWCNTs might possess good affinity for bone tissue and promote bone formation when added to a scaffold consisting of BMP and a collagen sheet [14]. Thereafter, we attempted to elucidate the mechanism of enhanced bone tissue regeneration by CNTs. In 2011, we showed that CNTs served as seeds for the crystallization of hydroxyapatite, the major component of bone, and that CNTs attracted Ca ions to activate osteoblasts. In the same study, we found that alkaline phosphatase released by osteoblasts caused hydroxyapatite to deposit around CNTs [39]. Based on these findings, we believe that CNTs can serve as a functional scaffold for bone formation and promote the process of bone tissue regeneration by interaction with the living body.

Data shown in Table 1 are the Young's modulus, measured using a four point bending apparatus. On the other hand, a compression analysis (Fig 2) showed that MWCNT blocks have a higher Young's modulus than bones. From this result, it is concluded that MWCNT blocks have a higher compression strength but relatively weak bending strength. MWCNT blocks showed higher protein adsorption ability and loading efficiency, and released more adsorbed protein than collagen on day 0 and 1. BSA release is reported to be similar in quantity to BMP release [40]. In the mouse ectopic bone formation model, rhBMP-2 loaded MWCNT blocks showed better bone formation than collagen sheets. Our results suggest that MWCNT blocks effectively retain and release rhBMP-2 in the location of bone formation.

The MWCNT blocks used in this study ensured good cell proliferative activity. The smaller difference in cell count found on Day 3 than on Day 1 is attributable to the shorter time needed to reach confluence on the MWCNT block and thereby to the slowing of proliferation. MC3T3-E1 cells are used to evaluate cellular adhesion and proliferation on a wide variety of scaffold materials including polymers, hydroxyapatite, metals, etc. In our experiments, cell adherence to the MWCNT scaffold occurred earlier than than that to PET-reinforced collagen fibers. Many past studies have found that materials with biocompatible, nanostructured surfaces are more effective in promoting cell proliferation than conventional materials [41][42]. Our MWCNT block had a macroscopically flat surface with nanosize to microsize surface irregularities, that likely favored cell proliferative activity [43][44][45].

MWCNT blocks also showed an increase in ALP activity regardless of the presence of rhBMP-2. Shimizu et al. reported that MWCNTs stimulate osteoblast-like cells and induce bone calcification [46]. MC3T3-E1 cells have a typical osteoblastic morphology when cultured in alpha-MEM for 21 days [30]. Our data suggested that MWCNT blocks could stimulate MC3T3-E1 preosteoblasts and cause osteoblastic changes.

We believe that the MWCNT scaffold promotes osteogenesis by allowing osteoblasts to adhere to, and proliferate on, the MWCNT block surface, and that it serves as a filler even after osteogenesis is complete.

In this study, we for the first time demonstrated that MWCNTs alone can serve as a scaffold for BMP, and that their effectiveness as scaffolds for bone formation is comparable to that of collagen sheets, which are deemed the gold standard BMP-containing scaffolds [47]. As shown in Fig 2 and Table 1, the mechanical strength of MWCNT blocks is at a reasonable level, although not comparable to that of bone cortex. Thus, MWCNT blocks can serve as an adequate filler for bone defects and as bone formation inducer comparable to collagen sheets. In addition, as shown in Fig 8, the close contact between MWCNT blocks and newly formed bone is suggestive of their greater effectiveness as scaffolds for bone tissue regeneration. As such, MWCNT blocks are a completely new material with the potential to reinvigorate the field of BMP-based bone regenerative medicine, which is currently stagnant for lack of new clinical alternatives.

MWCNTs should not be applied clinically before carefully assessing their biosafety. To date, MWCNTs have been reported to be toxic to a range of cells [48]. Macrophages are reported to dissolve MWCNTs, but the speed of dissolution is slow [49] and MWCNTs might remain in the body semipermanently. However, their toxicity may have been ignored, as we reported before [50][51]. It is generally believed, however, that MWCNTs are not cytotoxic unless they are internalized by such cells [52]. The MWCNT blocks used in this study released almost no MWCNTs in the aqueous phase and the sloughing of CNTs from blocks was minimal, as they had been sintered into solid form. For this reason, internalization of MWCNTs by cells is considered quite unlikely. In our previous study, we found that the human alveolar macrophage cell line THP-1 released much less TNF-α, an inflammatory cytokine, after exposure to the same MWCNT than after exposure to the synthetic lipoprotein FSL-1 [53]. Phagocytosis of only a small amount of MWCNT by macrophages results in release of only a small amount inflammatory cytokine. In this study, *in vitro* testing showed that the MWCNT blocks were not toxic to mouse preosteoblasts. However, further biosafety studies, including long-term biokinetics, will be needed before MWCNT blocks can be applied clinically.

## Supporting information

S1 Fig Permission to republish. A copy of the copyright holder’s permission to republish the data described in Table 1.
(PDF)
