# Supplementary material for: A three-dimensional block structure consisting exclusively of carbon nanotubes serving as bone regeneration scaffold and as bone defect filler
Source: PLoS One. 2017 Feb 24;12(2):e0172601. doi: 10.1371/journal.pone.0172601 (PMC5325283; doi:10.1371/journal.pone.0172601)
Supplement: S1 Fig — A copy of the copyright holder’s permission to republish the data described in Table 1. (PDF) [file pone.0172601.s001.pdf]

Copyright Clearance Center RightsLink®

Home | Create Account | Help | Live Chat

ACS Publications
Most Trusted. Most Cited. Most Read.

Title: Super-Robust, Lightweight, Conducting Carbon Nanotube Blocks Cross-Linked by De-fluorination

Author: Yoshinori Sato, Makoto Ootsubo, Go Yamamoto, et al

Publication: ACS Nano

Publisher: American Chemical Society

Date: Feb 1, 2008

Copyright © 2008, American Chemical Society

LOGIN

If you're a copyright.com user, you can login to RightsLink using your copyright.com credentials. Already **a RightsLink user** or want to learn more?

Quick Price Estimate

Permission for this particular request is granted for print and electronic formats, and translations, at no charge. Figures and tables may be modified. Appropriate credit should be given. Please print this page for your records and provide a copy to your publisher. Requests for up to 4 figures require only this record. Five or more figures will generate a printout of additional terms and conditions. Appropriate credit should read: "Reprinted with permission from {COMPLETE REFERENCE CITATION}. Copyright {YEAR} American Chemical Society." Insert appropriate information in place of the capitalized words.

If credit is given to another source for the material you requested, permission must be obtained from that source.

- Number of Table/Figure/Micrographs is a required field. Please make a selection.

**I would like to...**	reuse in a Journal
**Requestor Type**	Non-profit
**Portion**	Table/Figure/Micrograph
**Number of Table/Figure/Micrographs**	Table 1, Figure 2
**Format**	Print and Electronic
**Select your currency**	JPY - ¥
**Quick Price**	Click Quick Price

QUICK PRICE | CONTINUE

This service provides permission for reuse only. If you do not have a copy of the article you are using, you may copy and paste the content and reuse according to the terms of your agreement. Please be advised that obtaining the content you license is a separate transaction not involving Rightslink.

Note: Individual Scheme and Structure reuse is free of charge and does not require a license. If the scheme or structure is identified as a Figure in the article, permission is required.

To request permission for a type of use not listed, please contact the publisher directly.

Copyright © 2015 Copyright Clearance Center, Inc. All Rights Reserved. Privacy statement. Terms and Conditions.
Comments? We would like to hear from you. E-mail us at customercare@copyright.com
